# The therapeutic potential of C-peptide in kidney disease: a protocol for a systematic review and meta-analysis

**DOI:** 10.1186/2046-4053-3-43

**Published:** 2014-05-02

**Authors:** James Shaw, Partha Shetty, Kevin Burns, Greg Knoll

**Affiliations:** 1 Department of Medicine, Division of Nephrology, Ottawa Hospital Research Institute, University of Ottawa, 1967 Riverside Drive, Ottawa, Ontario K1H7W9, Canada

**Keywords:** C-peptide, Proinsulin, Chronic kidney disease, Diabetes, Systematic review

## Abstract

**Background:**

Kidney disease remains a major cause of morbidity and mortality in Canada and worldwide. New medical treatments are needed to reduce the progression of kidney disease to improve patient outcomes. C-peptide is normally released by pancreatic beta-cells along with insulin in healthy individuals, and has been shown to have intrinsic biological activity and to potentially be renoprotective. The effect of exogenous C-peptide on kidney structure and function, and the role of C-peptide in the treatment of kidney disease have not yet been fully elucidated.

**Methods/Design:**

We will conduct a systematic review of the literature in human clinical trials and mammalian experimental models to ascertain the current evidence for the role of C-peptide as a potential therapeutic agent for the treatment of kidney disease. We aim to identify whether exogenously delivered C-peptide has an effect on clinically relevant outcomes such as glomerular filtration rate, proteinuria, kidney histology, requirement of renal replacement therapy, and mortality. We will search MEDLINE, EMBASE, and the Cochrane Central Databases for human or animal studies in which C-peptide was administered and renal endpoints were subsequently measured. Study quality will be assessed using the Cochrane Collaboration’s tool for assessing risk of bias. If appropriate, a meta-analysis will be performed as per standard techniques.

**Discussion:**

The results of this study will determine the potential role of C-peptide as a therapeutic intervention for patients with kidney disease and will help guide subsequent clinical trials. The study may also provide insight into which patients or disease states are likely to benefit the most from C-peptide.

**Systematic review registration:**

PROSPERO CRD42014007472

## Background

Chronic kidney disease (CKD) is a growing international healthcare problem. Approximately 13% of North Americans are estimated to have CKD, a prevalence that is higher than populations in Europe, Asia, and Australia [1,2]. The presence of CKD is associated with increased morbidity, mortality, and healthcare costs [3]. Current clinical guidelines recommend a multifactorial approach to CKD, targeted towards risk factor and lifestyle modification, optimal control of blood pressure and blood sugar, reduction in proteinuria, and management of the metabolic sequelae of CKD [4,5]. Although a major objective of this clinical management strategy is to slow or prevent progression to end-stage renal disease (ESRD), this is unavoidable in many cases and results in the requirement for renal replacement therapies, which are associated with adverse outcomes [6]. Thus, there is a need for additional therapies for the management of kidney diseases to not only reduce the risk of progression to ESRD, but improve patient outcomes overall.

A central and accepted paradigm for insulin biosynthesis is the transcription and translation of the *INS* gene from chromosome 11 that generates a 110 amino acid polypeptide termed preproinsulin [7]. The subsequent post-translational modification of this molecule results in an intermediate molecule called proinsulin, which is then processed and split into insulin and C-peptide that are both released into the circulation in equimolar amounts from pancreatic beta cells [8-10]. C-peptide was initially thought of as a necessary yet biologically inert by-product of this process, but has retained clinical utility as a semi-quantitative marker of insulin secretion [10]. As examples, measurement of circulating C-peptide levels is useful in the management of diabetic patients to determine residual beta cell function, in pancreas or islet cell transplant patients to determine graft function, and in the workup of patients with hypoglycemia.

In contrast to the clinical role of C-peptide as a marker of endogenous insulin secretion, there are studies that support biological activity of C-peptide [11]. Importantly, some studies have suggested that C-peptide has renoprotective properties. Observational studies of patients with diabetes mellitus type 1 or 2 have correlated higher C-peptide levels with decreased prevalence of microvascular complications including diabetic nephropathy [12-15], reviewed in [16]. Furthermore, patients with type 1 diabetes have shown improved renal function following pancreas transplant, a procedure that repletes both C-peptide and insulin from transplanted beta cells [17,18]. Finally, small trials in which C-peptide was administered to experimental subjects with type 1 diabetes have also shown that C-peptide may improve renal function in these patients independent of any potential effect on glycemic control [19,20]. However, the therapeutic potential of C-peptide for patients with diabetic kidney disease remains incompletely understood. Whether the potential benefit of C-peptide is limited to patients with diabetes, or is applicable to a broader group of patients is currently unknown.

## Methods/design

### Rationale, objectives, and type of studies

The purpose of this systematic review is to synthesize available data from human and animal experiments, specifically examining the impact of exogenous C-peptide on markers of kidney function compared to control, without the limitation of a particular etiology of kidney disease.

### Information sources and search strategy

The electronic databases MEDLINE, EMBASE, and the Cochrane Central databases will be searched using standard controlled vocabulary (MeSH or EMTREE), text words, and keywords. The search will be intentionally broad to be as sensitive as possible and not to miss any relevant studies (see Appendix 1 for the full search strategy). An information specialist with previous systematic review experience will be consulted regarding the search strategy.

### Article selection

All titles and abstracts resulting from our initial search will be screened independently by two reviewers. Titles without abstracts will have the full text reviewed unless the article can be clearly excluded based on the information provided. Following reconciliation of differences between reviewers, the full text of the selected articles will be completely screened by each reviewer independently. During this process, a final decision for inclusion or exclusion will be made according to the criteria below. Any discrepancies will be resolved by a third party.

### Inclusion and exclusion criteria

Peer-reviewed published articles will be included if they meet all of the following criteria:

1. The experimental subjects are either humans or other mammals of any age;

2. The study intervention involves the administration of exogenous C-peptide to subjects;

3. The reported outcomes are related to relevant markers of kidney function, kidney disease, requirement for renal replacement therapy, or mortality; and

4. The manuscript must be written in English.

Studies using purified, artificial, recombinant, synthetic, or long acting formulations of C-peptide will be included for analysis. C-peptide from any species will be acceptable for inclusion.

Single case reports, narrative reviews, and studies reporting exclusively *in vitro* cell culture experiments, or *ex vivo* experiments will be excluded. We will also exclude studies in which animals or humans were given C-peptide, but the results contain only cellular or molecular endpoints such as change in gene expression profile, change in protein or enzyme concentration or activity, or cellular viability. There will be no limits on study publication date, other study design parameters, or sample size.

### Data collection process

Full text of all included articles will be electronically saved and each article will be assigned a unique code. Reference lists will be manually reviewed for other potentially eligible articles. In each included article, we will use a standardized form to collect information pertaining to:

1. Study design, methods, and timing of C-peptide administration;

2. Characteristics of experimental subjects;

3. C-peptide type and dosing;

4. Relevant renal outcomes such as glomerular filtration rate (GFR), serum creatinine, proteinuria/albuminuria, hematuria, renal blood flow, urine electrolyte excretion, kidney size, requirements for renal replacement therapy, and kidney histologic parameters;

5. Pertinent non-renal outcomes such as HbA1c, hemodynamic parameters (including blood pressure), cardiovascular risk, stroke risk, and mortality;

6. Study funding sources; and

7. Information necessary for risk of bias assessment.

### Quality assessment

Any randomized controlled trials that are included will be assessed for bias using the Cochrane Collaboration’s tool for assessing risk of bias.

### Data synthesis and analysis

Human and animal studies will be analyzed separately. Additionally, because it is anticipated that kidney function in diabetes will be the most well studied model among the search results, diabetic nephropathy studies will be analyzed separately from studies looking at other types of kidney disease.

We anticipate several sources of heterogeneity among our final dataset, particularly among the animal studies since we expect variation in disease model employed, methodology including experimental timing, and differences in chosen outcomes of interest. In particular, the C-peptide dose, route, and duration of administration may vary considerably between studies. For studies that have comparable methodology and outcomes, we will pool the results using a random-effects model for continuous or categorical variables as appropriate. In situations where data cannot be pooled, a narrative synthesis will be provided.

The current evidence for a therapeutic role of C-peptide in the context of kidney disease will be summarized based on the results of the search. The potential future clinical use of C-peptide will be discussed, along with suggested further research directions.

## Discussion

The aim of this systematic review is to ascertain the current evidence for and against the notion that exogenous delivery of proinsulin C-peptide has a potential beneficial effect on parameters of renal function. The rigorous and systematic nature of our review will ensure that it includes the best available information. Effort will be made to identify and limit procedural sources of bias and data heterogeneity.

Because the therapeutic use of C-peptide is still in an early experimental stage we have purposely decided to keep the search strategy and study inclusion criteria relatively broad in order to capture a comprehensive understanding of the potential therapeutic use for C-peptide in patients with kidney disease of any etiology. Although it is anticipated that most included studies will focus on models of CKD, we will not exclude studies looking at the role of C-peptide in the treatment of acute kidney injury (AKI) if they exist, so long as they meet eligibility criteria.

Our review may be limited by the number of available studies in the literature. As such, we have intentionally included both human and other mammalian studies to determine if a biological effect or signal for C-peptide truly exists. The added benefit of this is that other mechanisms of kidney injury may be tested against C-peptide administration in animal models that have not been considered for human trial at present, and may identify further areas of research.

We have elected to exclude studies in which C-peptide was given to whole animals, but reported results pertain only to changes in gene or protein expression, enzyme activity, or other cellular or molecular endpoints. The rationale for this exclusion is two-fold. First, we only wish to include studies that directly examine gross organ structure and function *per se*, to facilitate clinical interpretation of the potential therapeutic utility of C-peptide. In the context of this review we view genetic and molecular-based endpoints as mechanistic in nature. Although these studies serve the important role of furthering our understanding of the underlying processes leading to changes in organ structure and function, that level of detail is outside the scope of our research question. Second, inclusion of these studies would increase the heterogeneity of the pooled results since we anticipate different investigators will be looking at different genes, proteins, and signal transduction pathways. Overall, our decision to look at only human and *in vivo* mammalian studies was made to make the results of our study applicable to clinical practice and future clinical research as much as possible.

All medical interventions carry some degree of risk. Accordingly, our data extraction will also include relevant non-renal endpoints pertaining to hemodynamics (for example, blood pressure), glycemic control, cardiovascular risk, stroke risk, and mortality, if reported, to assess for any evidence of adverse events with C-peptide administration.

The results of this study will determine the potential role of C-peptide as a therapeutic intervention for patients with kidney disease, which will help guide subsequent clinical trials. The study may also provide insight into which patients or disease states are likely to benefit the most from C-peptide.

### Appendix 1: Search strategy

Database: Embase Classic + Embase <1947 to 2014 January 17>, Ovid MEDLINE(R) In-Process & Other Non-Indexed Citations and Ovid MEDLINE(R) <1946 to Present > Search Strategy:

1. c-peptide/(20016)

2. c-peptide.tw. (21593)

3. or/1-2 (27211)

4. exp kidney diseases/(1123898)

5. kidney disease$.tw. (81271)

6. glomerulonephritis.tw. (54594)

7. nephropath$.tw. (95394)

8. nephrosclerosis.tw. (3115)

9. nephritis.tw. (44363)

10. nephrotoxic$.tw. (39827)

11. glomerulosclerosis.tw. (16781)

12. renal insufficiency.tw. (46280)

13. ((impair$ or damage$ or injur$) adj2 (renal or kidney)).tw. (95922)

14. exp proteinuria/(100535)

15. proteinuria.tw. (67668)

16. exp kidney function tests/(76360)

17. (glomerular filtration rate or glomerulus filtration rate or GFR).tw. (74821)

18. blood urea nitrogen.tw. (16036)

19. ((kidney or renal) adj3 (renogra$ or angio$ or scinti$)).tw. (22169)

20. exp Kidney/(720592)

21. (kidney size or kidney mass).tw. (2038)

22. ((small$ or large$) adj2 kidne$).tw. (6099)

23. exp biopsy/and (kidney or renal).tw. (56542)

24. ((kidney or renal) adj2 biopsy).tw. (28834)

25. or/4-24 (1767311)

26. 3 and 25 (1952)

27. 26 use prmz (727)

28. c-peptide/(20016)

29. c-peptide.tw. (21593)

30. or/28-29 (27211)

31. exp kidney disease/(1123898)

32. kidney disease$.tw. (81271)

33. glomerulonephritis.tw. (54594)

34. nephropath$.tw. (95394)

35. nephrosclerosis.tw. (3115)

36. nephritis.tw. (44363)

37. nephrotoxic$.tw. (39827)

38. glomerulosclerosis.tw. (16781)

39. renal insufficiency.tw. (46280)

40. ((impair$ or damage$ or injur$) adj2 (renal or kidney)).tw. (95922)

41. exp proteinuria/(100535)

42. proteinuria.tw. (67668)

43. exp glomerulus filtration rate/(53592)

44. (glomerular filtration rate or glomerulus filtration rate or GFR).tw. (74821)

45. blood urea nitrogen.tw. (16036)

46. exp kidney function/(167997)

47. exp kidney size/(1888)

48. exp kidney mass/(3215)

49. exp kidney examination/(61135)

50. exp kidney/(720592)

51. ((kidney or renal) adj3 (renogra$ or angio$ or scinti$)).tw. (22169)

52. (kidney size or kidney mass).tw. (2038)

53. ((small$ or large$) adj2 kidney$).tw. (6099)

54. ((kidney or renal) adj2 biopsy).tw. (28834)

55. or/31-54 (1797601)

56. 30 and 55 (2165)

57. 56 use emczd (1469)

58. 27 or 57 (2196)

59. limit 58 to english language (2005)

60. remove duplicates from 59 (1560)

Database: EBM Reviews – Cochrane Database of Systematic Reviews <2005 to December 2013>, EBM Reviews – ACP Journal Club <1991 to December 2013>, EBM Reviews – Database of Abstracts of Reviews of Effects <4th Quarter 2013>, EBM Reviews – Cochrane Central Register of Controlled Trials < December 2013>, EBM Reviews – Cochrane Methodology Register <3rd Quarter 2012>, EBM Reviews – Health Technology Assessment <4th Quarter 2013>, EBM Reviews – NHS Economic Evaluation Database <4th Quarter 2013>

Search Strategy:

1. c-peptide.mp. (1810)

2. kidney disease$.mp. (3604)

3. glomerulonephritis.mp. (892)

4. nephropath$.mp. (3940)

5. nephrosclerosis.mp. (64)

6. nephritis.mp. (572)

7. nephrotoxic$.mp. (1466)

8. glomerulosclerosis.mp. (148)

9. renal insufficiency.mp. (1751)

10. ((impair$ or damage$ or injur$) adj2 (renal or kidney)).mp. (2725)

11. proteinuria.mp. (2142)

12. (glomerular filtration rate or glomerulus filtration rate of GFR).mp. (3171)

13. blood urea nitrogen.mp. (891)

14. ((kidney or renal) adj3 (renogra$ or angio$ or scinti$)).mp. (713)

15. kidney.mp. (19171)

16. (kidney size or kidney mass).mp. (18)

17. ((small$ or large$) adj2 kidney$).mp. (43)

18. ((kidney or renal) adj2 biopsy).mp. (286)

19. or/2-18 (25977)

20. 1 and 19 (82)

21. limit 20 to english language [Limit not valid in CDSR,ACP Journal Club,DARE,CCTR,CLCMR; records were retained] (82)

22. remove duplicates from 21 (82)

## Abbreviations

AKI: Acute kidney injury; CKD: Chronic kidney disease; ESRD: End-stage renal disease; GFR: Glomerular filtration rate.

## Competing interests

The authors declare that they have no competing interests.

## Authors’ contributions

JS conceived of the study. The review was designed by JS, PS, and GK. JS drafted the manuscript. GK and KB provided content and methodological expertise, feedback, and critical comments on the overall study design, protocol, and manuscript. All authors read and approved the final manuscript.
